# Per-Vaginal Enucleation of the Uterus, &c.

**Published:** 1880-06

**Authors:** L. C. Lane


### PER-VAGINAL ENUCLEATION OF THE UTERUS, WITH SPONTANEOUS CLOSURE OF THE PERITONEAL OPENING WITHOUT SUTURE.

BY L. C. LANE, M. D.

About the 1st of February, I was consulted by a lady from Stockton, affected with an epithelial cancer, seated in the neck of the uterus. The history was the usual one of pain in the pelvic region, excessive menstrual flow, &c., for which she had consulted Dr. F. W. Todd, some time previously. Having become satisfied that the case was one of malignant disease, and one only to be met by surgical means, Dr. Todd advised and performed ablation of the affected portion of the cervix. Recovery was rapid, and for a time, it seemed probable that the disease had been cured. About the last of January, however, signs of recurrence manifested themselves, when through the advice of Dr. Todd, the lady was sent to this city. On examination, I concurred in the diagnosis of epithelioma, which it should have been stated, had been verified by a leading microscopist, and I advised enucleation of the entire organ.

This operation was done at Stockton early in February, by myself and Drs. Todd, Ruggles, Eddy, Phillips and Farnum, in the following manner: The woman being placed on her side, the posterior vaginal wall being retracted by Sim's speculum, and the uterus being prolapsed by traction with Pean's tenaculum forceps, an opening was made through the fold of Douglas, when the fundus uteri was caught with the extracting forceps, and made to so revolve about its transverse axis that the Fallopian tubes and ovaries were brought low down in the pelvic excavation in such a manner that the base of the tubes and accompanying arteries became accessible and easily ligated. Ligation was done with strong silken cord, so passed through buttonholes in the broad ligaments that they could not afterwards slip off. This portion of the operation was completed in fifteen minutes, but the detachment of the organ from the bladder was long and tedious, but finally successfully done, without opening that viscus; yet so thin was the remaining vesical wall that the luster of the catheter which served as a guide, at times could be seen. The organ being removed, the pelvic excavation was rinsed out with a one per cent. solution of carbolic acid; a Nelaton flexible catheter was placed in the bladder; the pelvic excavation was filled with lint saturated with four per cent. carbolized linseed oil, and the abdomen covered with India rubber ice-bags. A drainage tube was so fixed alongside of the carbolized lint as to allow the escape of any fluids which should be passed out from the wounded surface. Under this mode of treatment, convalescence proceeded uninterruptedly towards recovery, which happy event was greatly favored by the assiduous and intelligent watching of the patient by Dr. Todd.

This, my second enucleation of the uterus per vaginam, differs from the first in this, that here the peritcneal cavity was freely opened, so much so that more than once during the operation the omentum protruded and had to be returned; and afterwards, no attempt was made to close the breach left in the peritoneum.

Though, as Don Quixotte says, "one swallow does not make a summer," yet I think it will be allowed that it is something of an approach towards it; and hence, from the results thus obtained, enucleation per vaginam may claim precedence equally with, or even over decollation, scooping out and supra-pubic ablation, for the treatment of uterine epithelioma. For decollation leaves a part of the affected internal mucous membrane behind, since the disease rapidly climbs up to the fundus on the inside. Scooping is like one blindfolded trying to catch an enemy whose eyes are open; and the traumatic assault upon the organism in the abdominal section is much greater than that where removal is effected per vias naturales.

Besides the advantages just offered, a brief excursion into the domain of physics furnishes a few more in behalf of this mode of removal. Thus, when the dynamic energy of respiratory work is estimated, we find that two-thirds of it, to wit, the inspiratory effort, is favorable to drainage; and even during expiration the resistance of the intestinal gases hermetically closes the abdominal cavity. To the favorite method of scooping, to which Sims, the greatest of living gynecologists, has recently given such an impetus, the objection may be offered that the truncated vessels remain in the solid uterine tissue as so many doors wide ajar, through which the fugitive bacteria, monads, panum's poison, or what not, can find unchallenged ingress. But in the mode pursued in the above case, the wounded surface remaining is loose and relaxed, favorable to contraction of the blood vessels, and unfavorable to lymphatic circulation, as the experiments of Ludwig in regard to sanguineous and lymphatic circulation have demonstrated.

March 6th. Through Dr. Farnum, who has just visited Stockton, I learn that the patient has so far recovered that Dr. Todd, the attending physician, has discontinued his visits; also that after the lapse of the first week, the vaginal tampon and drainage tube were dispensed with.—*Pacific Med. and Surg. Journal.*

